# Electrospun Nb-doped TiO_2_ nanofiber support for Pt nanoparticles with high electrocatalytic activity and durability

**DOI:** 10.1038/srep44411

**Published:** 2017-03-14

**Authors:** MinJoong Kim, ChoRong Kwon, KwangSup Eom, JiHyun Kim, EunAe Cho

**Affiliations:** 1 Department of Materials Science and Engineering, Korea Advanced Institute of Science and Technology (KAIST), 291 Daehak-ro, Yuseong-gu, Daejeon, 34141, Republic of Korea; 2 Department of Energy Environment Policy and Technology, Green School, Korea University, 145 Anam-ro, Seongbuk-gu, Seoul, 02841, Republic of Korea; 3 Fuel Cell Research Center, Korea Institute of Science and Technology (KIST), 5 Hwarang-ro 14-gil, Seongbuk-gu, Seoul, 02792, Republic of Korea; 4 School of Materials and Engineering, Gwangju Institute of Science and Technology (GIST), 123 Cheomdangwagi-ro, Buk-gu, Gwangju, 61005, Republic of Korea

## Abstract

This study explores a facile method to prepare an efficient and durable support for Pt catalyst of polymer electrolyte membrane fuel cell (PEMFC). As a candidate, Nb-doped TiO_2_ (Nb-TiO_2_) nanofibers are simply fabricated using an electrospinning technique, followed by a heat treatment. Doping Nb into the TiO_2_ nanofibers leads to a drastic increase in electrical conductivity with doping level of up to 25 at. % (Nb_0.25_Ti_0.75_O_2_). Pt nanoparticles are synthesized on the prepared 25 at. % Nb-doped TiO_2_-nanofibers (Pt/Nb-TiO_2_) as well as on a commercial powdered carbon black (Pt/C). The Pt/Nb-TiO_2_ nanofiber catalyst exhibits similar oxygen reaction reduction (ORR) activity to that of the Pt/C catalyst. However, during an accelerated stress test (AST), the Pt/Nb-TiO_2_ nanofiber catalyst retained more than 60% of the initial ORR activity while the Pt/C catalyst lost 65% of the initial activity. The excellent durability of the Pt/Nb-TiO_2_ nanofiber catalyst can be attributed to high corrosion resistance of TiO_2_ and strong interaction between Pt and TiO_2_.

Polymer electrolyte membrane fuel cell (PEMFC) is an electrochemical energy conversion device to produce electricity from hydrogen and oxygen. The PEMFC has advantages of high efficiency and environmental friendliness and attracts much attention as a power source for homes, buildings and transportations. However, the high fabrication cost and short lifespan are still major obstacles for commercialization. Among the various components of the PEMFC, electrode materials play a substantial role in determining the cost and durability. Therefore, development of well-performing and durable electrode materials has been a main concern in the PEMFC research area.

The most widely used electrode material for PEMFC is Pt nanoparticles supported on powdered carbon black (Pt/C), which can significantly reduce Pt usage compared with unsupported Pt black. As a support material, carbon has merits of low cost and high electrical conductivity^1^ and demerits of corrosion and low durability^2^. Particularly during start-up and shut-down of a PEMFC stack, carbon supports in the cathode are subjected to a severe corrosion environment via the following reaction^2^:


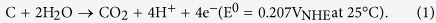


As carbon supports corrode to form carbon dioxide, Pt nanoparticles originally sitting on the carbon supports collapse and agglomerate^3^,^4^. Loss of the Pt catalysts due to carbon corrosion leads to drastic performance decay^5^,^6^,^7^. The cathode carbon corrosion is known as one of the crucial sources for degradation of PEMFC, particularly in automotive applications^8^. Therefore, in order to enhance durability of PEMFC, it is strongly required to replace carbon with the material which has high electrical conductivity and high corrosion resistance under the fuel cell operating conditions^9^.

As candidate support materials, conductive or semi-conductive metal oxides such as ITO^10^, SnO_2_^11^,^12^, WO_x_^13^,^14^,^15^, and TiO_x_^16^,^17^,^18^,^19^,^20^,^21^ have been studied. In addition to the excellent corrosion resistance, those materials are expected to enhance stability of Pt catalyst owing to the strong surface interaction between metal oxides and Pt nanoparticles^15^. Among those metal oxides, titanium oxide (TiO_2_) has attracted consideration as a novel support material due to its stability in the fuel cell operation condition, low cost, commercial availability and the ease of controlling its shape and structure. However, since pure TiO_2_ is a semiconductor with a band gap energy of 3.2 eV (anatase) and 3.0 eV (rutile)^22^, its electrical conductivity has to be enhanced to be used as a catalyst support material.

Doping of n-type dopants, whose atomic radius is similar to that of Ti, can increase the electrical conductivity of TiO_2_. Nb, which exhibits a pentavalent ionic state, is the most commonly used n-type dopant for TiO_2_^23^,^24^,^25^. It was reported that Nb-doped TiO_2_ (Nb-TiO_2_) has sufficient electrical conductivity for catalyst support material and Pt catalysts supported on Nb-TiO_2_ exhibit enhanced durability compared with those on the commercial carbon^26^,^27^,^28^,^29^,^30^. In most of the previous studies, Nb-TiO_2_ was synthesized using solution-based processes such as the hydrothermal^27^, sol-gel route^28^,^29^ and template-assisted multiple-step^26^,^30^. However, these methods have low product yield and consist of complicated procedures possibly leading to low reproducibility.

In this study, we aimed to develop a facile and scalable fabrication method for Nb-TiO_2_ support for Pt catalyst with high electrochemical stability and high electrical conductivity. So, we synthesized Nb-TiO_2_ nanofibers using the electrospinning technique, which is known as a facile, cost-effective, and scalable method for the synthesis of metal oxide nanofibers^31^. Moreover, the anisotropic 1-D structure of electrospun nanofiber is suitable for a catalyst support due to its high surface area^32^. To attain high electrical conductivity, the synthesis procedure of Nb-TiO_2_ nanofibers was examined with regard to the calcination temperature and Nb doping level. Electrocatalytic activity and durability of Pt catalyst supported on the Nb-TiO_2_ nanofibers were evaluated in comparison with Pt catalyst supported on a powered carbon black.

## Results

### Synthesis of Nb-TiO_2_ nanofibers

**Figure 1:**
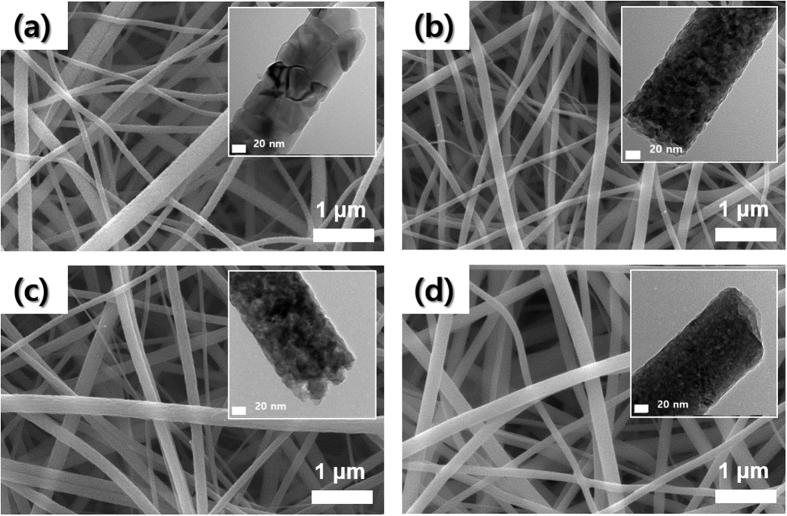
SEM and TEM images of electrospun Nb-TiO_2_ nanofibers with various Nb contents (after calcination in air at 700 °C for 1 h): (**a**) 0 at. %, (**b**) 10 at. %, (**c**) 25 at. %, (**d**) 50 at. %.

TiO_2_ and Nb-TiO_2_ nanofibers were obtained by calcining as-spun nanofibers in air at 700 °C for 1 h. The calcination condition was optimized based on synthesis of pure TiO_2_ nanofibers as shown in Figs S1–3. At 700 °C, anatase was fully transformed into stable rutile (Fig. S2). Fig. 1 (a–d) present SEM and TEM images of the TiO_2_ and the Nb-TiO_2_ nanofibers with Nb doping levels of 10, 25, and 50 at. %. Nb doping level was defined as an atomic ratio of Nb to (Nb + Ti). As shown in SEM images, all of the samples have a similar and uniform 1-D nanostructure of approximately 100 nm in diameter. Ti and Nb ions are also uniformly distributed throughout the nanofibers irrespective of Nb content (Fig. S4). However, Nb-TiO_2_ nanofibers have smaller crystallites than the TiO_2_ nanofiber as demonstrated in TEM images (Fig. 1 inset images).

**Figure 2:**
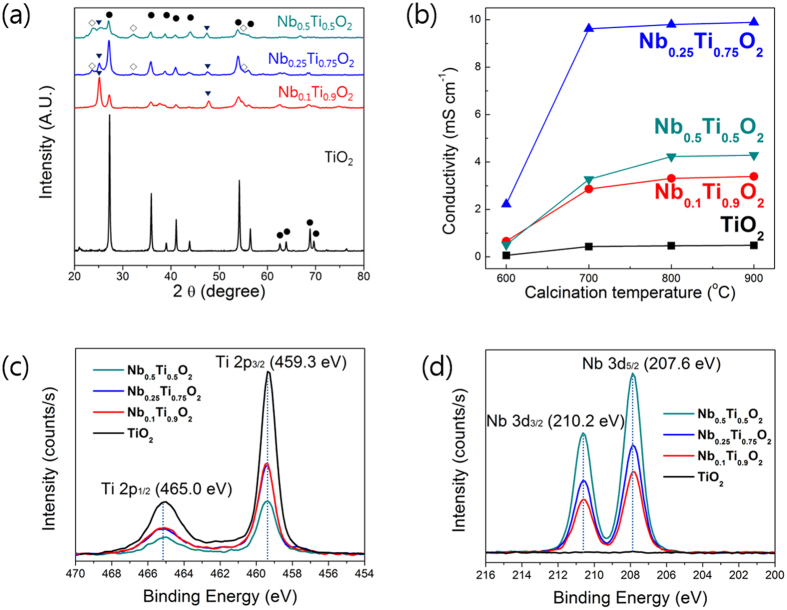
(**a**) X-ray diffraction patterns of Nb-TiO_2_ nanofibers with different doping levels of Nb; • rutile, ▾ anatase, ⋄ Nb oxides. (**b**) Effects of calcination temperature and Nb contents on electrical conductivity of Nb-TiO_2_ nanofibers. (**c**) Ti 2p and (**d**) Nb 3d XPS spectra of TiO_2_ and Nb-TiO_2_ nanofibers.

Figure 2(a) present XRD patterns for the TiO_2_ and the Nb-TiO_2_ nanofibers. Changes in crystal structure by Nb doping were observed in the XRD patterns; i) peak intensities of the Nb-TiO_2_ nanofibers were significantly lower than those of the TiO_2_ nanofibers, ii) anatase phase appeared in the Nb-TiO_2_ nanofibers unlike the TiO_2_ nanofiber with perfect rutile. The decrease in peak intensity by Nb doping resulted from the reduction in crystallite size as observed in Fig. 1. Presence of anatase phase in the Nb-TiO_2_ nanofibers can be associated with Nb ions. It was reported that doped Nb ions hinder diffusion of Ti ions required for crystal growth and anatase-rutile phase transformation^33^,^34^, leading to the decrease in the crystallite size and the appreance of anatase phase in the Nb-TiO_2_ nanofibers.

Compared to the TiO_2_ nanofiber, 10 at. % Nb-TiO_2_ nanofiber exhibited anatase peaks very clearly. However, with increasing Nb content from 10 to 25 at. %, anatase peaks diminished. When Nb doping level increased further to 50 at. %, both of rutile and anatase peaks diminished with increase in Nb oxide peaks, such as Nb_2_O_5_. It is known that Nb ions can be expelled from TiO_2_ when Nb doping exceeds a certain level^33^. Thus, anatase-rutile phase transformation can resume in the 25 at. % Nb-doped TiO_2_ nanofibers, which causes the decrease in anatase peak intensity. 50 at. % Nb-TiO_2_ could be a composite with a large amount of Nb-oxides due to increased expulsion of Nb ions. These results are in a good agreement with the previous results on the anatase-to-rutile phase transition of TiO_2_ by Nb doping^33^.

Figure 2(b) shows the effect of the Nb doping level and calcination temperature on the electrical conductivity of the Nb-TiO_2_ nanofibers. With increasing Nb content from 0 to 25 at. %, the electrical conductivity greatly increased reflecting that 25 at. % Nb-TiO_2_ nanofiber contains higher doping level of Nb than in 10 at. % Nb-TiO_2_. However, the electrical conductivity was lowered with 50 at. % Nb content due to the formation of a large amount of Nb-oxides as observed in the XRD patterns^32^. The formation of Nb-oxides from expelled Nb ions could lower the Nb doping efficiency into TiO_2_ phase, which lead to the decrease in electrical conductivity.

To understand the contribution of Nb doping to electrical conduction of TiO_2_, Ti 2p and Nb 3d spectra of 0, 10, 25, and 50 at. % Nb-TiO_2_ nanofibers were measured by XPS as shown in Fig. 2(c) and (d). All of the Nb-TiO_2_ nanofibers have the same peak position of Ti 2p_3/2_ at 459. 3 eV corresponding to Ti^4+^ valence and Nb 3d_5/2_ peak at 207.2 eV corresponding to Nb^5+^ valence state regardless of Nb doping level. These results imply that when Nb^5+^ ions substitute for Ti^4+^ ions, charge compensation occurs by creation of one Ti cation vacancy per four Nb ions rather than by reduction of Ti^4+^ to Ti^3+^, as described in the following equation^35^:


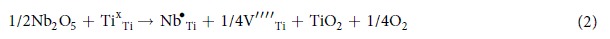


Thus, by Nb-doping up to 25 at. %, Ti cation vacancies are formed in TiO_2_ crystalline nanofibers and contribute to the significant increase in electrical conductivity. Based on those results, Pt nanoparticles were synthesized on the 25 at. % Nb-TiO_2_ nanofibers and on a commercial Vulcan carbon.

### Electrochemical characterization

**Figure 3:**
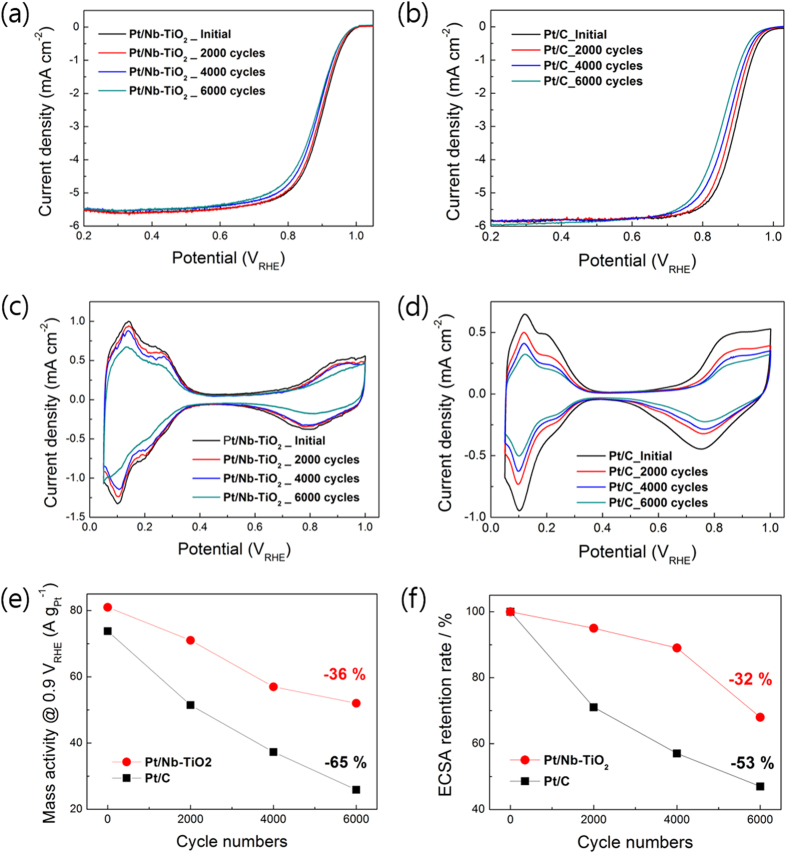
ORR polarization curves and CVs measured during the AST: (**a**), (**c**) Pt/Nb-TiO_2_ nanofiber catalyst and (**b**), (**d)** Pt/C catalyst. (**e**) mass activity @ 0.9 V_RHE_ and (**f**) ECSA retention rate of Pt nanoparticles as a function of cycle number for Pt/Nb-TiO_2_ nanofiber catalyst and Pt/C catalyst.

Figure 3(a–d) shows the oxygen reduction reaction (ORR) and the cyclic voltammograms (CVs) polarization plots for Pt/Nb-TiO_2_ nanofiber catalyst and Pt/C catalyst before and after an accelerated stress test (AST). The ORR polarization plots were measured in O_2_-saturated 0.1 M HClO_4_ solution at a scan late of 5 mV s^−1^ with a rotating speed of 1600 rpm. The CVs were measured in Ar-saturated 0.1 M HClO_4_ solution at a scan rate of 20 mV s^−1^. As an AST, voltage cycling was conducted repetitively between 0.6 and 1.1 V_RHE_ in an O_2_-saturated 0.1 M HClO_4_ solution at a scan rate of 50 mV s^−1^.

Before the AST, there is little difference in the ORR activity between the Pt/Nb-TiO_2_ nanofiber catalyst and the Pt/C catalyst. Half-wave potentials obtained from each ORR polarization curve are also almost same; 0.896 for the Pt/Nb-TiO_2_ nanofiber and 0.895 V_RHE_ for the Pt/C catalyst. To precisely compare the initial electrocatalytic activity of the Pt/Nb-TiO_2_ nanofiber catalyst and the Pt/C catalyst, we calculated the kinetic current density at 0.9 V_RHE_. The kinetic current density (*i*_*k*_) is expressed as Eq. (3). *i* is the measured current density at 0.9 V_RHE_, and *i*_*l*_ is the limiting current density. The calculated kinetic current density was normalized by Pt loading on the glassy carbon to obtain the mass activity of Pt. The Pt loading was same for both catalysts; 60 µg_pt_cm^−2^.


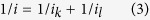


The initial mass activity of the Pt/Nb-TiO_2_ nanofiber catalyst at 0.9 V_RHE_ (81 A g_pt_^−1^) was slightly higher than that of the Pt/C catalyst (73.8 A g_pt_^−1^). It is well established for metal-oxide supported catalysts that strong metal-support interactions (SMSI) can enhance catalytic activity and durability of the Pt catalysts toward ORR^14^,^36^. Those results demonstrate that the Pt/Nb-TiO_2_ nanofiber catalyst exhibits excellent ORR activity comparable with the Pt/C catalyst, and the Nb–TiO_2_ nanofiber has sufficient conductivity and surface area to serve as a catalyst support.

During the AST, ORR activity also decreased with repeating voltage cycles as shown Fig. 3(a) and (b). It is notable that the decrease in ORR activity is much faster for the Pt/C catalyst than for the Pt/Nb-TiO_2_ nanofiber catalyst during the AST. Half-wave potential of the Pt/Nb-TiO_2_ nanofiber catalyst deceased only by 17 mV, whereas that of the Pt/C catalyst decreased by 40 mV during 6,000 cycles. The hydrogen adsorption/desorption peak area of CVs also gradually decreased with repeating voltage cycles as shown in Fig. 3(c) and (d), indicating a decrease in electrochemical surface area (ECSA) of Pt. The mass activity at 0.9 V_RHE_ and ECSA changes of each catalyst were calculated and plotted as a function of cycle number in Fig. 3(e) and (f). During 6,000 cycles, the mass activity of the Pt/Nb-TiO_2_ nanofiber catalyst measured at 0.9 V_RHE_ decreased from 81 to 52 A/g_pt_^−1^ while that of the Pt/C drastically decreased from 73.8 to 25.9 A g_pt_^−1^. The Pt/Nb-TiO_2_ nanofiber catalyst retained more than 60% of the initial ORR activity while the Pt/C catalyst lost almost 65% of the initial activity during the AST. During the same potential cycles, ECSA of the Pt/Nb-TiO_2_ nanofiber, which is calculated from the hydrogen adsorption/desorption peak, decreased by about 32%, whereas that of the Pt/C catalyst decreased by about 53% of the initial values. The Pt/Nb-TiO_2_ nanofiber catalyst has remarkably enhanced durability than the Pt/C catalyst.

**Figure 4:**
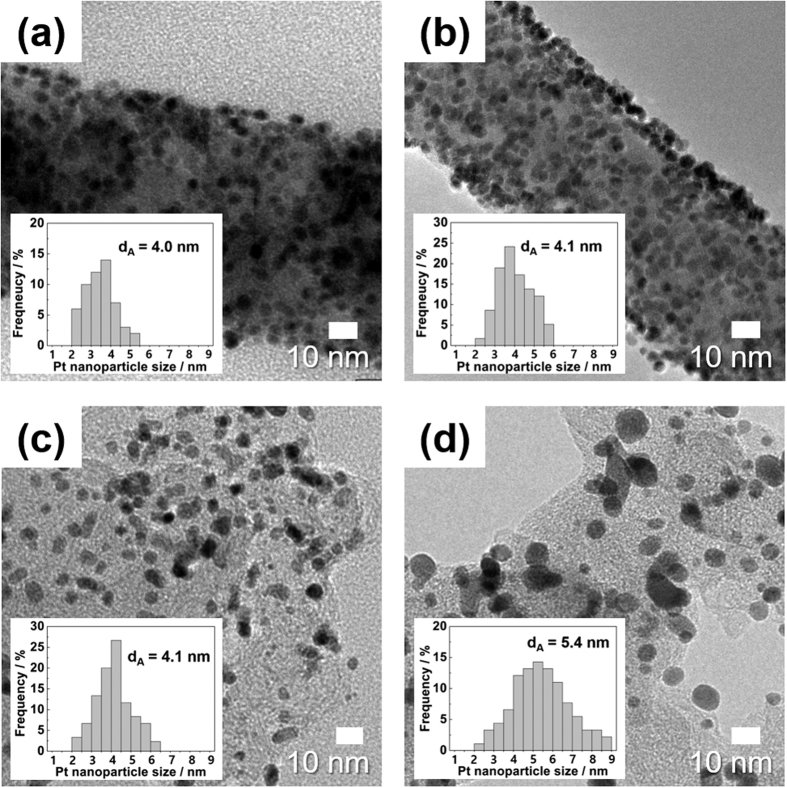
TEM images of Pt nanoparticles of (**a**), (**b**) the Pt/Nb-TiO_2_ nanofiber catalyst and (**c**), (**d**) the Pt/C catalyst before and after the AST.

Figure 4 presents TEM images of the Pt/Nb-TiO_2_ nanofiber catalyst and the Pt/C catalyst before and after the AST. For the Pt/Nb-TiO_2_ nanofiber catalyst, the Pt nanoparticles on the Nb-TiO_2_ nanofiber negligibly agglomerated and still uniformly distributed after the AST. In contrast, the Pt nanoparticles on the carbon support heavily agglomerated and the mean particle size increased to 5.4 nm from 4.1 nm during the AST. In addition to the agglomeration of Pt nanoparticles, significant amount of Pt nanoparticles on the carbon support could be dissolved, resulting in the significant losses in ORR activity and ECSA of the Pt/C catalyst as shown in Fig. 3^37^. The enhanced durability of the Pt/Nb-TiO_2_ nanofiber catalyst would be attributed to: (a) the higher corrosion resistance of the Nb-TiO_2_ nanofiber support compared with carbon at high potential in acidic environments and (b) the strong interaction between Pt and metal oxide support^14^,^36^. All of those results reveal that the Pt/Nb-TiO_2_ nanofiber has a large potential to be used as a durable catalyst to replace the Pt/C catalyst.

## Discussion

As an efficient and durable catalyst support material for polymer electrolyte membrane fuel cell (PEMFC), Nb-TiO_2_ nanofibers were simply synthesized using the facile electrospinning followed by the heat treatment. TiO_2_ nanofibers prepared by calcining the as-spun precursor solution at 700 °C in air atmosphere. Doping Nb into TiO_2_ leads to a drastic increase in the electrical conductivity up to 25 at. %. A further increase in the Nb content lowered the electrical conductivity owing to formation of a large amount of Nb oxides. Based on those results, Pt nanoparticles were fabricated on the 25 at. % Nb-doped TiO_2_ nanofibers calcined at 700 °C in air for 1 h and on a commercial Vulcan carbon for comparison.

Electrocatalytic activity and durability of those prepared catalysts were evaluated in terms of mass activity toward the ORR and ECSA. Pt/Nb-doped TiO_2_ nanofiber catalyst exhibited excellent ORR activity comparable with Pt/C catalyst (81 A g_pt_^−1^). Those results are the highest value among the TiO_2_-supported Pt catalysts up to date^16^,^17^,^18^,^19^,^20^,^21^,^26^,^27^,^28^,^29^. During 6,000 potential cycles between 0.6 and 1.1 V_RHE_, the mass activity measured at 0.9 V_RHE_ decreased from 81 to 52 A g_pt_^−1^ for the Pt/Nb-TiO_2_ nanofiber catalyst and from 73.8 to 25.9 A g_pt_^−1^ for the Pt/C catalyst, the ECSA of Pt/Nb-TiO_2_ nanofiber catalyst decreased by about 32% while that of the Pt/C decreased about by 53% of the initial values. Thus, it can be concluded the Nb-TiO_2_ nanofiber catalyst exhibit similar initial electrocatalytic activity but much higher durability compared with the Pt/C catalyst.

## Materials and Methods

### Preparation of Nb-TiO_2_ nanofiber support

To prepare an Nb-TiO_2_ precursor solution, 4 ml of acetic acid (≥99.7%), 2 ml of titanium tetraisopropoxide (TTIP, 97%), 2 g polyvinylpyrrolidone (PVP, M_w_ = 1,300,000), and appropriate amount of niobium ethoxide (99.95%) for 0, 10, 25, and 50 at. % Nb doping were added to 14 ml of ethyl alcohol (EtOH, anhydrous). Then, the prepared solutions were degassed by sonication for 5 min and transferred to an injector for electrospinning.

The Nb-TiO_2_ precursor solution was fed at a constant rate of 3 ml h^−1^ through a stainless steel needle with a flat outlet. Diameter of the needle was 27 G (200 μm). Distance between the tip of the needle and the collector plate was 10 cm, and applied voltage was 20 kV. Because of the external electrostatic field and electrostatic repulsion between the surface charges, a so-called ‘Taylor cone’ was formed at the tip of the needle instead of a droplet. During electrospinning, the effects of the surface tension were outbalanced by the electrostatic interactions, and a liquid jet was formed. The needle was slowly moved along two perpendicular paths to randomly deposit the TiO_2_ nanofibers on the aluminum foil, which was placed on top of the collector plate. The as-spun nanofibers were dried at room temperature for 15 hours and then calcined in air at 600, 700, 800, 900 °C, respectively, for 1 h to convert the TTIP into crystalline TiO_2_ and to completely remove PVP and remaining solvents.

### Synthesis of Pt catalyst on Nb-TiO_2_ nanofiber and carbon supports

Pt catalyst was synthesized on Nb-TiO_2_ nanofiber support (denoted Pt/Nb-TiO_2_ nanofiber) using the borohydride reduction method. Firstly, H_2_PtCl_6_·6H_2_O (Pt precursor) was dissolved in EtOH. The prepared Nb-TiO_2_ nanofiber support was dispersed in EtOH and then mixed with the prepared Pt precursor solution by mechanical stirring. Then, pH value of the mixed suspension was adjusted to 8.5 by adding 0.5 M KOH or 0.5 M HCl solution. The Pt precursor was reduced by adding excess amount of 0.1 M NaBH_4_ solution to the mixed solution and being stirred for 2 h. The prepared Pt/Nb-TiO_2_ nanofiber was washed with D.I. water and then dried at 40 °C for 24 h. For comparison, Pt catalyst was fabricated on a commercial Vulcan carbon (Pt/C) using the modified ethylene glycol method. Pt precursor solution was prepared as described above^27^. Pt loading on Nb-TiO_2_ nanofiber was 20 wt. %, and on Vulcan carbon was 40 wt. %. To adjust Pt particle size on Vulcan carbon to 4 nm, we had to increase Pt content to 40 wt. %. Pt content in the prepared samples was measured by inductively coupled plasma-mass spectroscopy (ICP-MS).

### Physical characterizations

To determine calcination temperature, thermogravimetric analysis (TGA) was conducted for the as-spun nanofibers from 20 to 900 °C at a heating rate of 10 °C min^−1^ under an air atmosphere. Electrical conductivity of the calcined TiO_2_ nanofibers was obtained from I-V measurement using a 2-point probe equipment as shown in Fig. S5. Crystal structure of the samples were analyzed from X-ray diffraction (XRD) (Bruker D-8 advance) spectra. The diffraction spectra were recorded using Cu-Kα (λ = 1.5046 Å) radiation (20 kV and 20 mA) from 2θ angles of 20° to 80° with a step size of 0.04° at a rate of 2° min^−1^.

Morphology of the samples was observed using environmental field emission-secondary electron microscopy (FE-SEM) and field emission-transmission electron microscopy (FE-TEM). Chemical composition of the Nb-TiO_2_ nanofibers was measured using the energy dispersive X-ray analysis (EDAX) detector in the SEM or TEM. From X-ray photoelectron spectroscopy (XPS), elemental composition, chemical state and electronic state of the elements were analyzed.

### Electrochemical characterization

Electrochemical experiments were performed in a conventional three-electrode cell. The working electrode was catalyst-coated glassy carbon. The counter electrode was a platinum wire and a saturated calomel electrode (SCE) was used as the reference electrode. All the potentials were represented on the scale of the reversible hydrogen electrode (RHE). To prepare the catalyst ink for the working electrode, 5 mg of Pt/C and 10 mg of Pt/Nb–TiO_2_ nanofiber catalysts were ultrasonically suspended in 800 μl of isopropyl alcohol (IPA) and 60 μl of Nafion solution (5 wt. % solution, Aldrich). Then, 5 μl of ink was transferred using a micropipette onto a glassy carbon rotating disk electrode (RDE, 5 mm diameter) and dried.

Cyclic voltammetry (CV) was carried out in the potential range between 0.05 and 1.0 V_RHE_ at a scan rate of 20 mV s^−1^ in Ar-saturated 0.1 M HClO_4_ solution. Electrochemical active surface (ECSA) was estimated based on the H_2_ desorption peaks observed at potentials from 0.05 and 0.35 V_RHE_. The hydrogen desorption charge was assumed to be 0.21 mC cm^−2^ 38.

Activity of the prepared catalyst for the oxygen reduction reaction (ORR) was measured in O_2_-saturated 0.1 M HClO_4_ solution at room temperature. The RDE rotating rate was 1600 rpm, and the sweep rate was 5 mV s^−1^. As an accelerated stress tests (AST), potential cycling was conducted in the potential range from 0.6 to 1.1 V_RHE_ in the O_2_-saturated 0.1 M HClO_4_ solution at a scan rate of 50 mV s^−1^.

## Additional Information

**How to cite this article:** Kim, M. *et al*. Electrospun Nb-doped TiO_2_ nanofiber support for Pt nanoparticles with high electrocatalytic activity and durability. *Sci. Rep.* **7**, 44411; doi: 10.1038/srep44411 (2017).

**Publisher's note:** Springer Nature remains neutral with regard to jurisdictional claims in published maps and institutional affiliations.

## Supplementary Material

Supplementary Information
