# Supplementary material for: Electrospun Nb-doped TiO2 nanofiber support for Pt nanoparticles with high electrocatalytic activity and durability
Source: Sci Rep. 2017 Mar 14;7:44411. doi: 10.1038/srep44411 (PMC5349578; doi:10.1038/srep44411)
Supplement: Supplementary Information [file srep44411-s1.pdf]

Supplementary Information

Electrospun Nb-doped TiO_2 nanofiber for Pt nanoparticles with high electrocatalytic activity and durability

MinJoong Kim[a,1], ChoRong Kwon[b,c,1], KwangSup Eom[d], JiHyun Kim[b], EunAe Cho[a,*]

[a]Department of Materials Science and Engineering, Korea Advanced Institute of Science and Technology (KAIST), 291 Daehak-ro, Yuseong-gu, Daejeon, 34141, Republic of Korea.

[b]Department of Energy Environment Policy and Technology, Green School, Korea University, 145 Anam-ro, Seongbuk-gu, Seoul, 02841, Republic of Korea

[c]Fuel Cell Research Center, Korea Institute of Science and Technology (KIST), 5 Hwarang-ro 14-gil, Seongbuk-gu, Seoul, 02792, Republic of Korea

[d]School of Materials and Engineering, Gwangju Institute of Science and Technology (GIST), 123 Cheomdangwagi-ro, Buk-gu, Gwangju, 61005, Republic of Korea

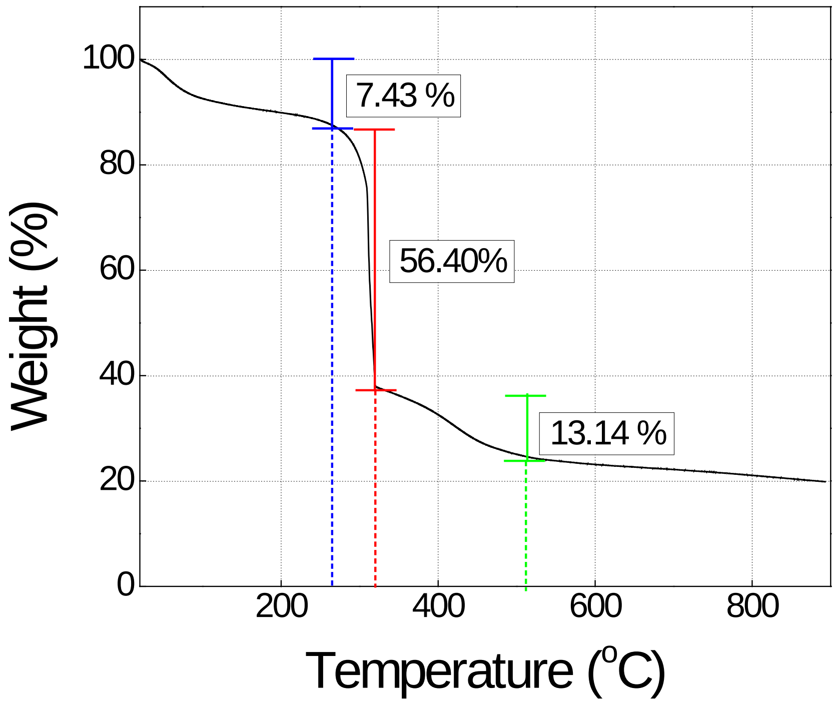

Fig. S1 TGA curve of as-spun nanofiber

Three regions of weight loss were observed; firstly, weight of 7.34 % loss occurred from 20 to 270 °C, resulting from evaporation of the solvents such as acetic acid, EtOH and water. Above 270 °C, polymer chains of PVP rapidly decomposed and evaporated, leading to a drastic weight loss (56.40 %) to 320 °C.[1] Above 320 °C, the remaining Ti precursor (TTIP) was oxidized to TiO_2 up to 500 °C in combination with dissociation of carbon and hydrogen. Above 500 °C, weight of the sample decreased very slowly, indicating that transformation of the precursor solution into TiO_2 is largely completed.

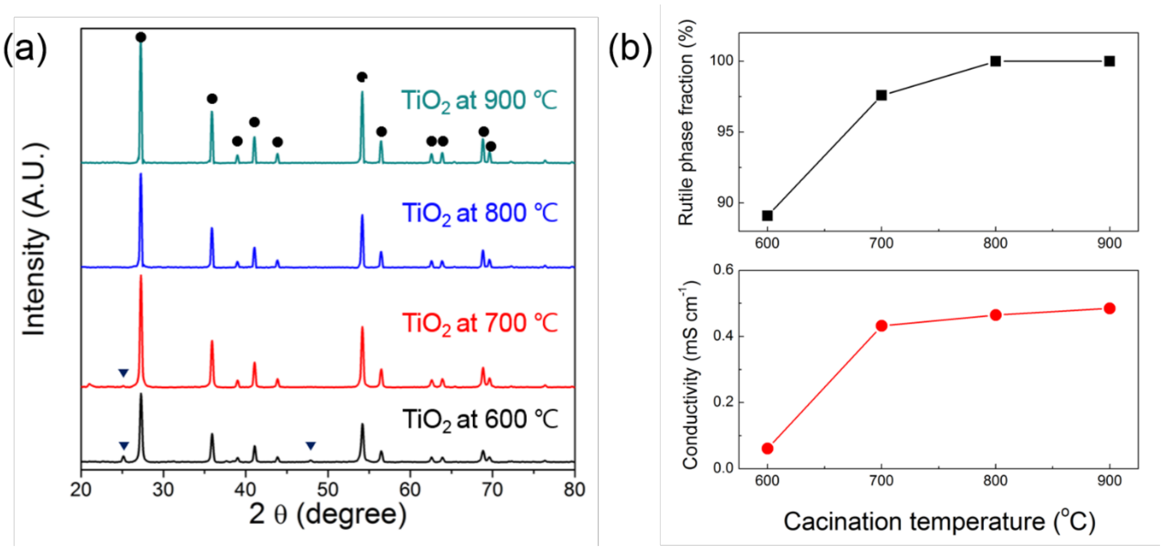

Fig. S2 (a) XRD patterns of electrospun TiO_2 nanofibers with different calcination temperatures: ● rutile, ▼anatase. (b) Effects of calcination temperature on rutile phase fraction and the electrical conductivity and of TiO_2 nanofibers.

As indicated in Fig. S2(a), only in the TiO_2 nanofibers calcined at 600 and 700 °C, small peaks from anatase phase were observed. Major phase in all the samples was rutile. Rutile phase fraction, which is defined as rutile/(anatase+rutile), can be estimated using the empirical relationship proposed by Depero et al.[2] The calculated fraction of rutile phase increased from 89.1 to 97.6 % with increasing the calcination temperature from 600 to 700 °C as presented in Fig. S2(b). TiO_2 nanofibers calcined at 800 or 900 °C exhibited almost 100 % of the rutile phase fraction with high crystallinity.

Band gap of the anatase and rutile phase is 3.2, and 3.0 eV, respectively.[3] The rutile with the lower band gap is electrically more conductive. Therefore, TiO_2 nanofibers calcined at a higher temperature and composed of a higher fraction of rutile phase, are expected to have higher electrical conductivity, as demonstrated in Fig. S2(b)

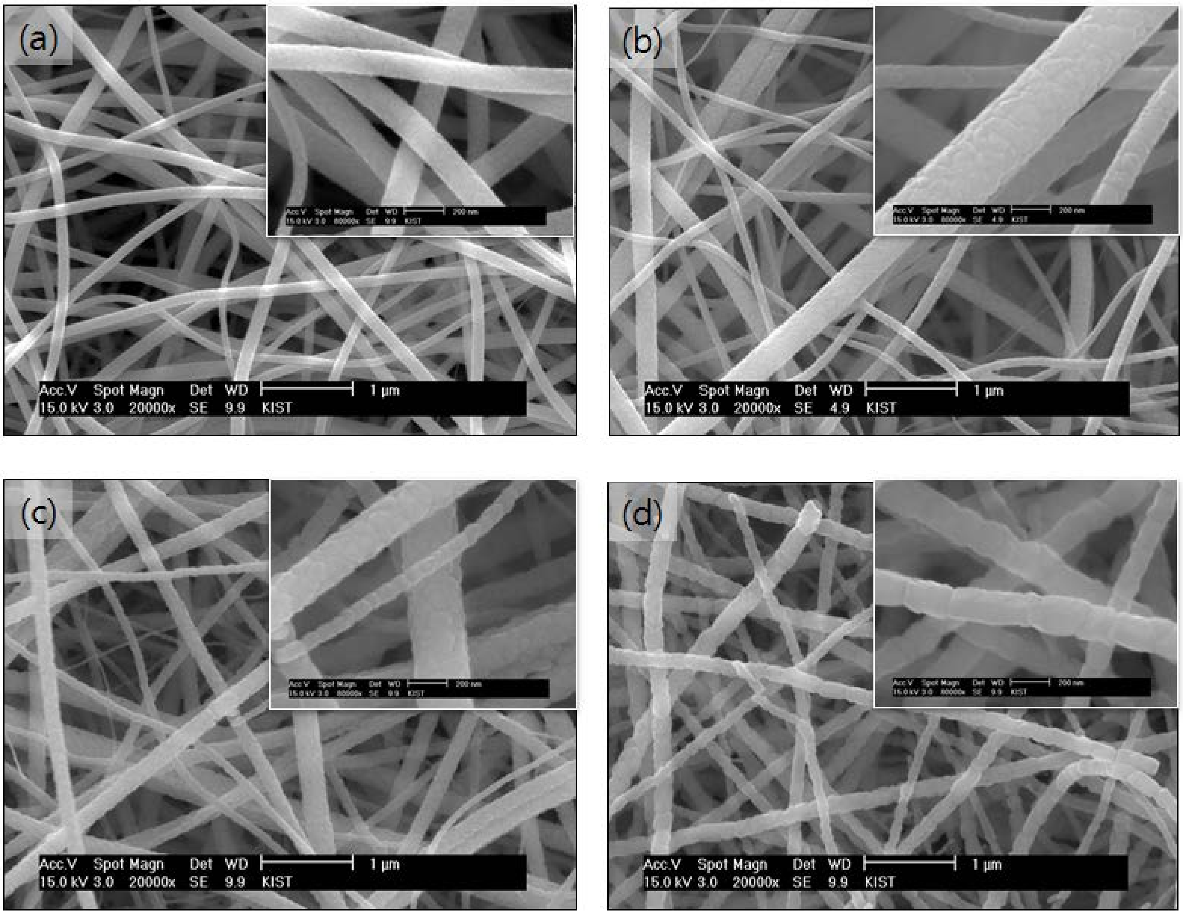

Fig. S3 SEM images of electrospun TiO_2 nanofibers with different calcination temperatures: (a) 600, (b) 700, (c) 800, (d) 900 °C

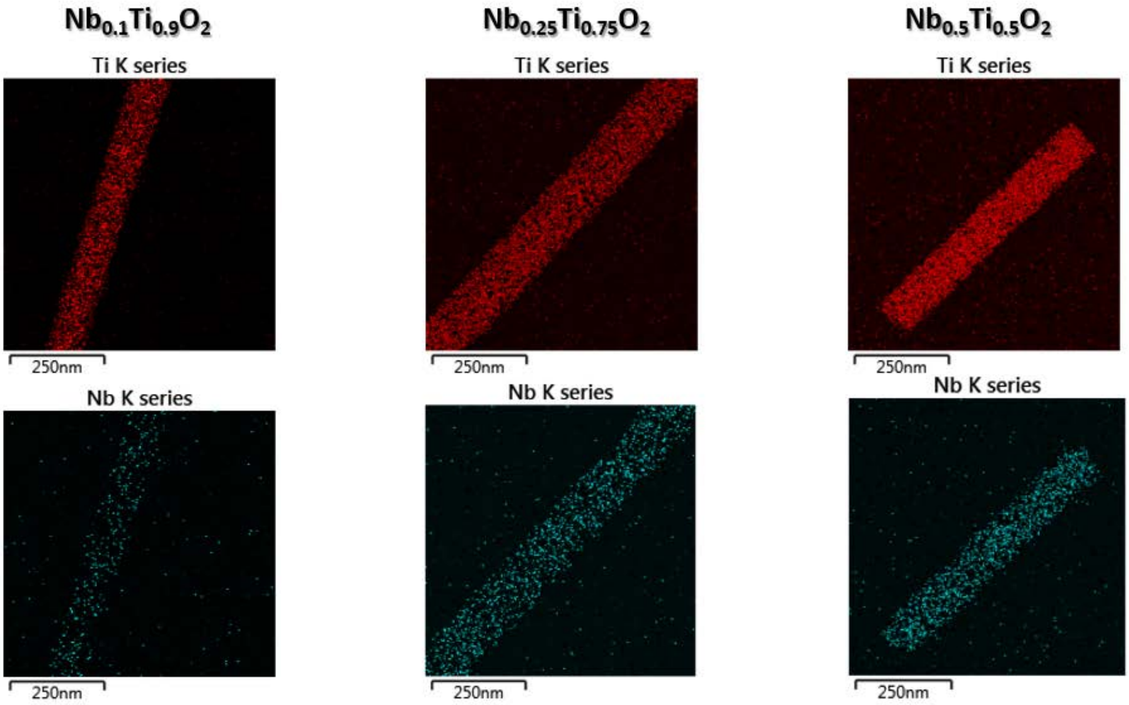

Fig. S4 EDS mapping images of Ti and Nb in the Nb-TiO_2 nanofibers with various Nb content.

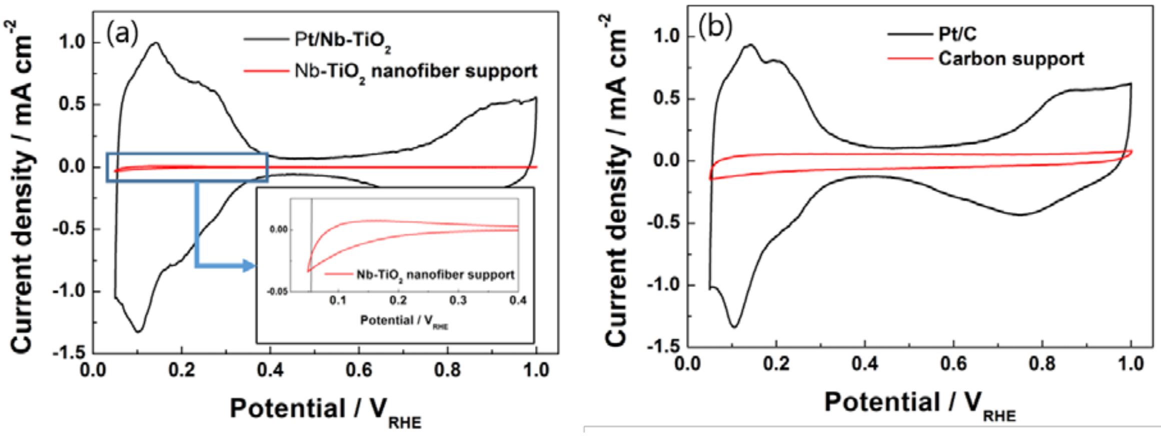

Fig. S5 CVs for (a) Pt/Nb-TiO_2 catalyst and Nb-TiO_2 support and (b) Pt/C catalyst and Vulcan carbon support in 0.1 M $HClO_4$ solution. Bare support loading was 240 μg cm^{-2}, and scan rate was 20 mV s^{-1}.

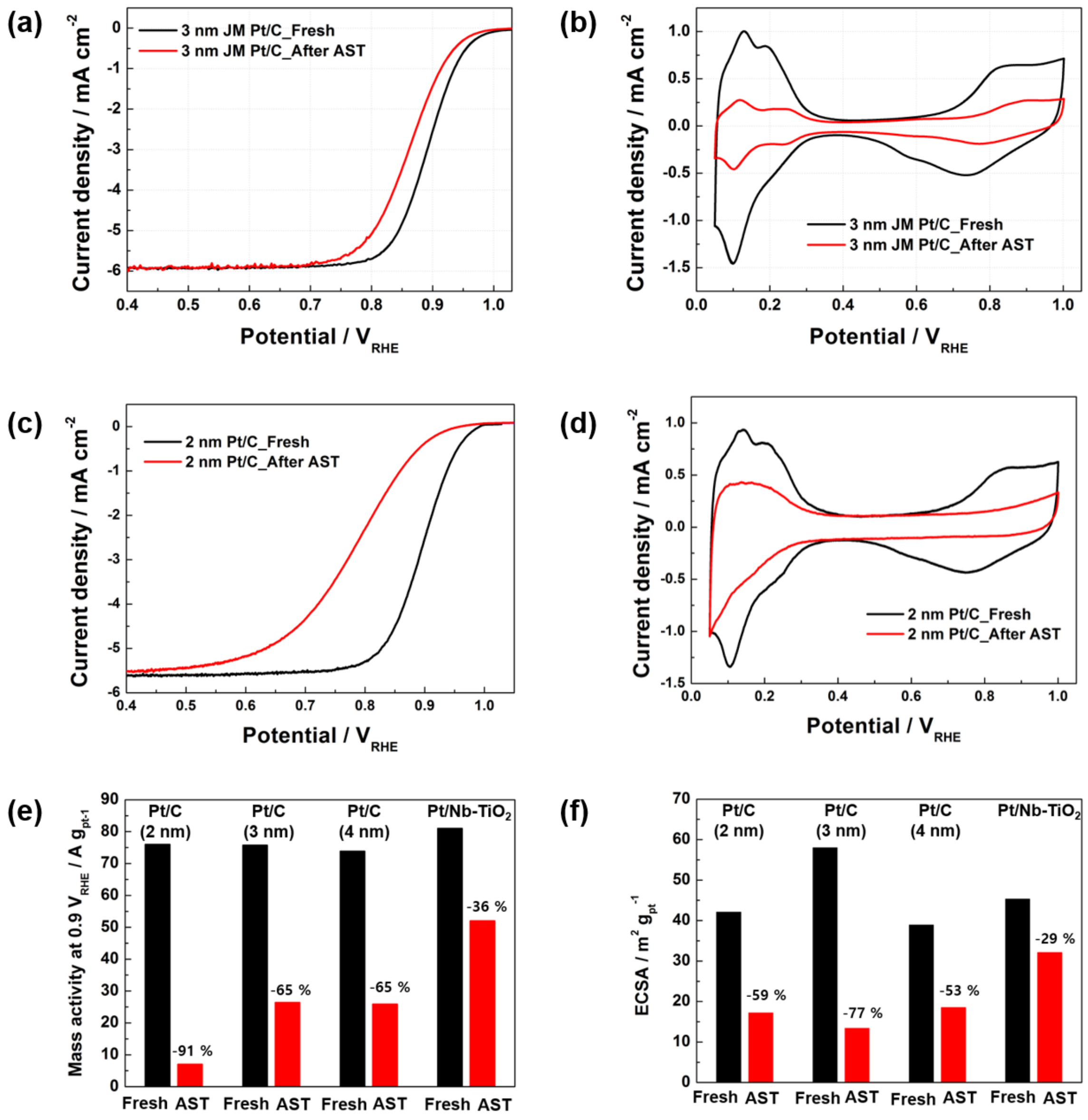

Fig. S6 (a) ORR polarization curves and (b) CVs of JM 3 nm Pt/C Pt/C catalyst, (c) ORR polarization curves and (d) CVs of home-made 2 nm Pt/C catalyst before and after the AST. (e) mass activity at 0.9 V_{RHE} and (f) ECSA changes of catalysts with different particle size during the AST.

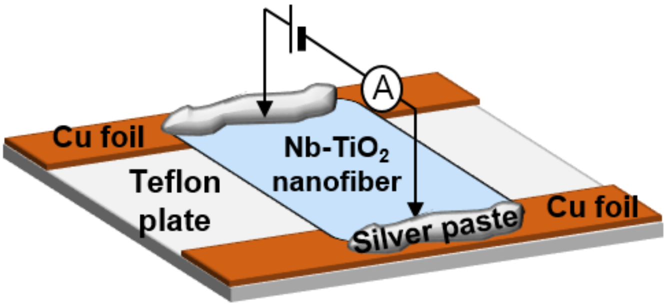

Fig. S7 Schematic illustration of the 2-point probe measurement.

Table 1 Previously-reported ORR mass activity at 0.9V$_{RHE}$ of TiO_2-supported Pt catalysts

**Catalysts**	**Mass activity at 0.9 V$_{RHE}$**
Pt/TiO_2-mesoporous carbon [4]	20 A g_{pt}^{-1}
Pt/Nb-TiO_2 (nanoparticle) [5]	70 A g_{pt}^{-1}
Pt/C-Ti [6]	50 A g_{pt}^{-1}
Our study (Pt/Nb-TiO_2 nanofiber)	81 A g_{pt}^{-1}

References

1. Mater. Sci. Eng. A, 2005, 398, 77
2. *J. Mater. Res.*, 1998, 13, 1644
3. *J. Phys. Chem., 2003, B* 107, 5709
4. Electrochim. Acta, 2010, 55, 8365
5. J. Power Sources, 2010, 195, 3961
6. Catal. Lett., 2010, 134, 288
